# Potential Mechanisms of Lactate Dehydrogenase and Bovine Serum Albumin Proteins as Antioxidants: A Mixed Experimental–Computational Study

**DOI:** 10.1155/bri/9638644

**Published:** 2025-02-10

**Authors:** Jing Ye, Amy Bounds, Madeline Crumpton, Mallory Long, Haley McDonough, Isabella Srikhirisawan, Shanzhen Gao

**Affiliations:** ^1^Department of Chemistry and Biochemistry, Salem College, Winston-Salem, North Carolina, USA; ^2^Department of Computer Information Systems, Virginia State University, Petersburg, Virginia, USA

## Abstract

Proteins have shown varying degrees of antioxidant activity. This study examined the potential mechanisms of interactions between proteins and radicals using chemical kinetics and computational methods. The study quantified the antioxidant activity of lactate dehydrogenase (LDH) and bovine serum albumin (BSA) through Trolox equivalent antioxidant capacity (TEAC) and oxygen radical absorbance capacity (ORAC) assays. BSA was about seven times and LDH 12 times more potent as antioxidants for 2,2′-azino-bis(3-ethylbenzothiazoline-6-sulfonate) (ABTS^•−^) than they were for peroxyl radicals. According to the evaluation of Trolox equivalents (TE) of 20 proteinogenic amino acids, tryptophan (with a TE value of 101 μmol TE/μmol) exhibited the highest antioxidant activity for ABTS^•−^, followed by tyrosine (38.7 μmol TE/μmol) and cysteine (30.5 μmol TE/μmol), lysine (0.193 μmol TE/μmol), arginine (0.0325 μmol TE/μmol), valine (0.0280 μmol TE/μmol), histidine (0.00689 μmol TE/μmol), and leucine (0.00560 μmol TE/μmol). The EC50 showed a similar order with a swap between valine and histidine. The antioxidant activity of the amino acids and proteins was temperature dependent. The rate laws, activation energy, and pre-exponential factor A of these reactions provided information on the reaction mechanisms, i.e., a biomolecular elementary step for the reaction of ABTS^•−^ with amino acids tryptophan, tyrosine, cysteine, or protein LDH, and a more complicated mechanism for BSA. The presence of –NH– or hydroxyl groups on aromatic rings enhanced the antioxidant ability of tryptophan and tyrosine. LDH's antioxidant activity did not affect its enzymatic activity, indicating that the radical reaction likely happened on the protein's surface without significantly altering its conformation. The molecular modeling and visualization showed potential reaction sites on the proteins' accessible tryptophan and tyrosine residues. However, the mere surface exposure of tryptophan and tyrosine does not guarantee their antioxidant activities.

## 1. Introduction

Reduction–oxidation (redox) reactions are common biochemical reactions in our bodies. Regulating these reactions is necessary to maintain cell viability and function [1]. Some redox reactions produce reactive oxygen species (ROS) or reactive nitrogen species (RNS), such as hydrogen peroxide or nitric oxide. Reactive species in the body can take on two forms: free radicals and nonradicals that can readily produce radicals. Although some radicals are necessary for natural cellular processes such as metabolism, gene expression, and signal transduction [2, 3], increasing ROS/RNS concentration above homeostatic levels can cause oxidative stress. High concentrations of ROS/RNS can potentially harm the body by damaging DNA, RNA, lipids, and proteins [1]. Oxidative stress has been linked to many disorders, including cancer, cataractogenesis, atherosclerosis, neurodegenerative diseases, diabetes, autism spectrum disorder, Down syndrome, and asthma [4, 5].

Various endogenous and exogenous factors can affect the ROS/RNS formation rate in the human body. Endogenous sources of ROS/RNS are produced in organelles with high oxygen consumption, such as mitochondria, peroxisomes, and the rough endoplasmic reticulum [2]. Exogenous sources of ROS/RNS include air pollution, cigarette smoking, radiation, certain drugs, and pesticides [3].

One way the body regulates ROS/RNS is through the work of antioxidants. Antioxidants are compounds that react with ROS/RNS and inhibit oxidations, thereby reducing the harmful effects of ROS/RNS in the body [6, 7]. While small-molecule antioxidants can be synthesized and optimized in the lab [8, 9], studies have identified various natural antioxidants, such as uric acid [10], bilirubin [11], vitamins A, C, and E [12], polyphenolic compounds [13, 14], and antioxidant proteins [4].

Medicinal plants containing antioxidant polyphenolic compounds have a long history of therapeutic application [15]. The elevation of antioxidants has been shown to mitigate cardiovascular disease, diabetes, metabolic syndrome, and accelerated aging [11, 16, 17]. However, the antioxidant compounds and their antioxidant activities must be retained during harvest, storage, and preparation [13, 14]. Thus, we need to understand the conditions, including pH and temperature, for processing antioxidants.

Certain proteins, such as superoxide dismutase [18], catalase [19], and glutathione peroxidase [20], enzymatically break down ROS/RNS and function as the first line of natural defense against the harmful effects of ROS/RNS in the body [21]. Furthermore, proteins and peptides that primarily perform different functions also show antioxidant activities [17]. However, some proteins showed considerable antioxidant activity and some did not. Researchers hope to predict antioxidant proteins based on their sequence [4, 22].

In 2017, researchers created the Antioxidant Protein Database (AOD) to organize the information discovered about known antioxidant proteins [23]. One such protein is bovine serum albumin (BSA), a serum albumin protein derived from cattle. BSA has a high capacity for binding with various ligands and acts as a transporter protein for endogenous and exogenous compounds, including ions, drugs, hormones, and fatty acids [7, 24].

Many antioxidant proteins, like BSA, are identified in the AOD, but little is known about the antioxidant capacity of lactate dehydrogenase (LDH). LDH is an enzyme that catalyzes the reversible conversion of pyruvate to lactate with the help of coenzyme NADH/NAD^+^ in anaerobic respiration [25, 26]. Although low levels of LDH are not considered harmful, a high level of LDH can indicate acute or chronic cell damage and several conditions such as stroke, cancer, heart attack, blood flow deficiency, hemolytic anemia, hepatitis, muscle injury, and tissue death [3, 27]. According to a study, LDH has been linked to oxidative stress and indirectly supports tumor survival by safeguarding the tumor from the effects of ROS/RNS [28]. However, the direct antioxidant activity of LDH and the mechanism by which it conducts antioxidant activity are unknown.

This research aims to investigate how BSA and LDH proteins have antioxidant capacity and explore where these reactions could occur in other proteins. The study examines the ability of LDH and BSA proteins and amino acids to neutralize reactive nitrogen and oxygen radicals through Trolox equivalent antioxidant capacity (TEAC), oxygen radical absorbance capacity (ORAC), and EC50 tests. Furthermore, the effect of temperature on the antioxidant activity of the proteins and amino acids was examined. Lastly, the study uses a computational protein-docking program and PyMOL to identify and visualize reactions between proteins (BSA and LDH) and free radicals and predict where a protein has the greatest potential to conduct antioxidant activity.

## 2. Materials and Methods

### 2.1. Preparation of TEAC Assay and Trolox Solution

The TEAC method was adapted from the study conducted by Re et al. [29]. Acros Organics was the source of Trolox, potassium persulfate, and 2,2′-azino-bis(3-ethylbenzothiazoline-6-sulfonate) (ABTS). To prepare the ABTS solution (0.015 M or 8.23 mg/mL), one 10 mg tablet of ABTS was dissolved in 1.215 mL of ultrapure water. To prepare potassium persulfate (0.005 M), 0.0135 g of potassium persulfate was dissolved with ultrapure water in a 10 mL volumetric flask. To prepare the ABTS^•+^ radical solution, 1 mL of 0.015 M ABTS was added to 1 mL potassium persulfate in a dark microtube. The resulting solution was vortexed for 1 min and stored at 4°C for at least 24 h before the kinetic experiment. A freshly made daily stock Trolox solution (2 mg/mL) was prepared by dissolving 0.010 g Trolox in 5 mL ethanol.

### 2.2. Preparation of ORAC Assay

Uranine and 2,2′-azobis (2-amidinopropane) dihydrochloride (AAPH) were purchased from Thermo Fisher Scientific. The procedure used in this experiment was adapted from Cao, Alessio, and Cutler [30]. Stock solutions of uranine (sodium fluorescein, 140 nM) and AAPH (240 mM) were prepared in 1x phosphate buffer saline (PBS). Trolox (0.01 g) was added to 5 mL ethanol to make a stock solution, which was later diluted with PBS. The final concentration of Trolox was determined using UV–vis spectroscopy. The antioxidant stock concentrations were 0.081 mg/mL Trolox, 9.3 mg/mL BSA, and 0.31 mg/mL LDH. The working Trolox concentration in this experiment was between 0.2 mg/L and 10 mg/L, BSA was less than 0.1 mg/mL, and LDH was less than 0.04 mg/mL. To ensure the total antioxidant capacity in 120 min, the protein concentration must be kept less than 0.1 mg/mL.

### 2.3. Preparation of Proteins and Amino Acids

BSA and bovine heart muscle LDH were obtained from Sigma-Aldrich, while rabbit muscle LDH was obtained from Calbiochem. To prepare the proteins for the kinetic experiment, each was dissolved in 1x PBS buffer to make stock solutions with a concentration of 13 mg/mL. The stock solutions were stored at 4°C for at least 24 h before the experiment. The molecular weight of LDH in the solution was evaluated using size-exclusion column chromatography.

Amino acids were acquired from Acros Organics, Sigma Life Science, or Alfa Aesar. Stock solutions of amino acids were prepared in 1x PBS with varying concentrations. The concentrations were as follows: 0.25 mg/mL Trp, 0.48 mg/mL Cys, 0.48 mg/mL Tyr, and 14 mg/mL Phe; 20 mg/mL Gly, Met, His, Val, Ile, Pro, Thr, Ala, Asn, Arg, Ser, Lys, and Gln; 10 mg/mL Leu; and 5.0 mg/mL Glu and Asp. The stock solutions were stored at 4°C and were used within 2 weeks.

### 2.4. Kinetic Study and Data Collection

In the TEAC experiment, the antioxidant was added to 0.075 mM ABTS^•−^ solution in PBS at room temperature (RT), and the mixture was immediately measured with a Thermo Scientific SPECTRONIC 200E spectrophotometer at 730 or 413 nm.

The kinetic study of ORAC assays was completed using the Synergy H1 microplate reader at an excitation wavelength of 485 nm and an emission wavelength of 520 nm. For 120 min, 81 readings were recorded at 90-s intervals.

### 2.5. Temperature Study

The reactant solutions were heated to specific temperatures and equilibrated for 7 minutes at the same temperature before being mixed. The initial rate of the reaction was then measured. The concentrations of antioxidants and radicals were kept constant while the temperature varied.

### 2.6. Evaluation of Radical Reaction on LDH Enzymatic Activity

The activity of the LDH enzyme was measured by observing the formation of NADH, which absorbs at 340 nm using a Thermo Scientific SPECTRONIC 200E spectrophotometer. Each assay contained 0.100 mL LDH (0.64 μM) and 2.900 mL cocktail, consisting of 1.900 mL CAPS (0.14 M, pH 10), 0.500 mL of lactate (0.15 M), and 0.500 mL of NAD^+^ (6 mM) [31]. The concentration of ABTS^•−^ used in the assay was either 0, 1, 2, or 3 μM.

### 2.7. Data Analysis

To determine the Trolox equivalent (TE) for each antioxidant at a given concentration, a linear fit was performed using the Trolox concentrations and the absorbance change (Δ*A*). The TE (μmol/L) was then divided by the antioxidant concentration (g/L) to calculate each sample's μmol TE/g protein.

We utilized the initial rate technique and the isolation method to determine the rate laws of the reactions. This method kept the concentration of either ABTS^•−^ radical or the antioxidant constant in a reaction. When determining the order of the reactions with respect to proteins or amino acids, ABTS^•−^ radical was in excess with a concentration of 40 μM. BSA had concentrations ranging from 0.3 to 1 μM, and LDH ranged from 0.5 to 7 μM. Trp concentration ranged from 0.5 to 5 μM, Tyr concentration ranged from 5 to 60 μM, and Cys concentration ranged from 3 to 20 μM.

The Arrhenius parameters were determined through a temperature study, where the natural logarithm of rate constants was graphed against the reciprocal of temperatures in Kelvin. The drawings for organic chemistry mechanisms were created using the KingDraw Professional Chemical Structure Editor.

Experiments were triplicated or duplicated, and the standard deviations were calculated with Microsoft Excel.

### 2.8. PyMOL

PyMOL 2.5 [32], a molecular visualization system written in C, C++, and Python, was used to identify and visualize the surface Tyr and Trp with a surface exposure area cutoff of 6.0–25.0 square Angstroms for Rabbit LDH (PDBID 3H3F) and BSA (PDBID 3V03).

### 2.9. Protein Docking

Protein docking experiments were performed using Chimera Version 1.15 [33] and AutoDock Vina [34]. ChimeraX [35] was also used to visualize protein structure and docking results. The protocol was adapted from Butt et al. [36]. The protein structures of LDH (PDB ID: 3H3F) and BSA (PDB ID: 3V03) were obtained from the Research Collaboratory for Structural Bioinformatics (RSCB) protein database. Small molecules such as ions, ligands, and inhibitors from the X-ray crystallography are often present in PDB structures. Therefore, all nonstandard residues and molecules were removed after importing the protein receptors into Chimera. LDH is a tetramer, but the PDB structures have a duplicate, so unnecessary protein chains were deleted. The protein receptors were then treated as rigid to facilitate docking simulations; according to the protocol, AutoDock Vina primarily treats receptors as rigid and ligands as flexible [37].

The ligands ABTS^•−^ and AAPH peroxyl radical (2-amidino-2-peroxylpropane cation) were built using the WebMO platform and then subjected to energy minimization. In this study, the ligands were treated as flexible with the number of active rotatable bonds in the simulation.

AutoDock Vina's receptor options were kept at default during the protein docking simulations. All ligand options, except for the “merge charges and remove lone pairs” option were also kept at default. The “merge charges and remove lone pairs” option was set to false to prevent the program from removing the lone electron present on the ligand. The maximum possible modes were set for the number of modes, exhaustiveness of search, and maximum energy difference.

Two docking methods were used to find the receptor area with the highest affinity for the ligands. In the first method, simulations were performed to divide the receptor into smaller sections and determine the area that was most likely to interact with the ligand. In the second method, the ligands were positioned near the surface Trp and/or surface Tyr because of their observed antioxidant activity.

During the analysis of the results, hydrogen bonds were set to be present. The lowest binding scores stood for the most stable interactions. Four main factors were considered when deciding whether an interaction has the potential to possess antioxidant activity. These factors include (1) the identity of the residues involved in the interaction, (2) whether the interaction site was near the nitrogen with a lone electron on the radical, (3) whether the interaction took place on the protein's surface, and (4) whether any other residues were present nearby that could be dimerized with the newly formed radical residue to neutralize it completely.

## 3. Results and Discussion

### 3.1. TEs of LDH, BSA, and Antioxidant Amino Acids

In the TEAC assay, ABTS radicals were created using a 3:1 ratio of ABTS to potassium persulfate. At least 24 h of reaction time were allowed to ensure the depletion of persulfate in the assay so that only radicals would react with antioxidants. ABTS^•+^ became ABTS^•−^ after diluting with 1x PBS solution, under which condition the two sulfonic acids were deprotonated. The 1x PBS buffer provides 0.01 M phosphate, 00027 M potassium chloride, and 0.137 M sodium chloride, and it has a pH of 7.4 at 25°C, closely matching the pH and total ion concentration of human fluids [38].

TEAC of bovine heart LDH (Bov LDH), rabbit muscle LDH (Rab LDH), and BSA were determined following a 5 min incubation with ABTS^•−^. In order to understand the mechanisms behind LDH and BSA's antioxidant activity, the antioxidant abilities of all 20 proteinogenic amino acids were also evaluated.  Table 1 lists the TEs for BSA, LDH, and various amino acids with significant antioxidant activities.

The ranking of amino acids' antioxidant abilities was found by comparing the μmol TE per μmol or μmol TE per gram. The ranking in terms of μmol TE per μmol was as follows: Trp > Tyr > (≈) Cys ≫ Lys > Arg > Val > His ≥ Leu. However, when comparing the same amino acids based on their μmol TE per gram, the ranking order for Tyr and Cys had been switched, and so had Arg and Val: Trp > Cys > (≈) Tyr ≫ Lys > Val > Arg > His ≥ Leu. Trp exhibited the highest antioxidant activity among amino acids. Like Cys, Tyr was more than 2 times less reactive than Trp in the presence of ABTS^•−^. BSA had a slightly higher antioxidant ability than LDH when using μmol TE per μmol but lower when using μmol TE per gram.

The total antioxidant capacity of Trolox, BSA, and LDH was also measured using the ORAC assay, which is different from the reactive nitrogen-based TEAC method. In this experiment, AAPH was used to induce the formation of a peroxyl radical (2-amidino-2-peroxylpropane hydrochloride) in the presence of uranine, a fluorescent probe. The decrease in fluorescence over time due to the radical was monitored in this reaction. The ability of a protein to inhibit the reaction of AAPH with uranine, thus curbing the decrease in fluorescence, was used to determine its total antioxidant scavenging activity. The ORAC assay only contains the fluorescent probe uranine and the radical generator AAPH in phosphate buffer. Uranine and proteins in the absence of AAPH as controls showed no significant change in fluorescence throughout the experiment, similar to the control of uranine with no protein and AAPH, eliminating the possibility of protein interference in fluorescence measurement. The TEs of BSA and LDH for the AAPH-generated peroxyl radical are listed in Table 2.

The results of our study show that BSA has a slightly higher potency than LDH as an antioxidant when eliminating the AAPH-generated peroxyl radical. Furthermore, when comparing their TE values, BSA exhibited seven times higher reactivity with ABTS^•−^ than with the peroxyl radical, while LDH showed 12 times higher reactivity with ABTS^•−^. The comparison could be more rigorous if the TE values in the TEAC method measure the total antioxidant ability like ORAC. These differences in antioxidant potency of the proteins toward reactive oxygen and nitrogen species could suggest different radical quenching mechanisms, perhaps due to the difference in reduction potentials of the radicals. It is worth noting that our protein docking experiment also indicated that the two radicals have different affinities for the proteins, and different residues may contribute to the antioxidant activities of the proteins for the two different radicals. We will discuss the details of this analysis in Section 3.6.

Although TEAC is commonly used to quantify the antioxidant capacity, it has certain limitations. Trolox is unstable and needs to be prepared daily, which is time-consuming and results in poor reproducibility. To ensure the experiment's accuracy and reproducibility, Trolox's extinction coefficient in 190 proof ethanol was determined to be 2800 L·mol^−1^·cm^−1^ or 11 L·g^−1^·cm^−1^ at 290.5 nm. Keeping LDH working concentrations below 200 mg/L is essential for the standard curve to ensure a linear regression. Therefore, the TE methods are only applicable to dilute LDH solutions.

TEs allow us to investigate the kinetic parameters by measuring the reaction's initial rate. In contrast, the ORAC and DPPH are good at estimating the total antioxidant capacity after some time (e.g., 30–120 min) but miss the initial rate of radical quenching. All those methods need a standard. Hence, the EC50 provides a convenient alternative to TEAC and ORAC for comparing antioxidant capacities without requiring Trolox or other standards.

### 3.2. EC50 of LDH, BSA, and Amino Acids at Various Time Points

The EC50 is the concentration of an antioxidant that reduces 50% of the radicals present in a given solution within a specific time frame. The EC50 for LDH, BSA, and amino acids at 1, 5, and 7 min were determined and are listed in Table 3.

In the study, all amino acids except for Val had similar rankings in antioxidant activity when evaluated using EC50 and TEAC methods. Trp displayed the most potent antioxidant activity among the amino acids, followed by Cys and Tyr. Lys, Arg, His, Val, and Leu exhibited significantly less potency in descending order. Lys was 100 times less potent than Tyr as an antioxidant. Arg, His, Val, and Leu were 10–50 times less potent than Lys. Gly, Met, and Phe showed slight quenching of ABTS^•−^ with negligible activities.

The EC50s of LDH and BSA monomers are similar to Trp and comparable to Tyr and Cys. Although each LDH monomer has six Trp, seven Tyr, and five Cys residues, and each BSA monomer has two Trp, 20 Tyr, and 35 Cys residues, our data suggests that only one equivalent Trp on the LDH monomer and approximately 3.3 ± 0.5 Tyr on the BSA monomer act as free radical quenchers. The antioxidant ability of the individual antioxidant residues in LDH and BSA is constrained in the protein. Likely, only antioxidant residues with certain conditions on the protein surface react with ABTS^•−^ with reduced activity.

Although the experiments were carried out at RT in PBS with a similar pH and ionic strength to that of body fluids in vivo, it is unknown how the proteins' antioxidant activities change, if at all, in vivo, where a much more complex environment and many other proteins are present. In addition, pH and the tertiary structure of proteins can vary in different cellular environments or compartments. Therefore, a pH study should be conducted before an in vivo study.

### 3.3. Rate Laws

**Figure 1:**
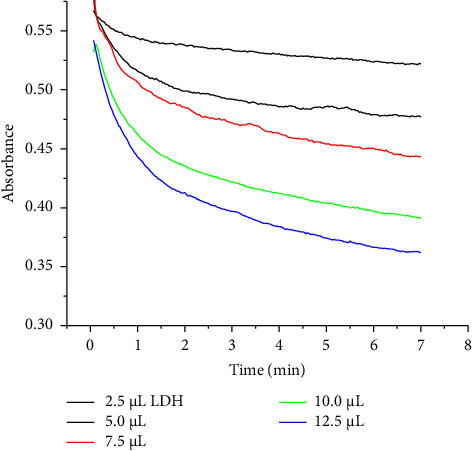
The change of absorbance over the first 7 min of the reaction of LDH with ABTS^•−^. The stock LDH concentration was 13 mg/mL. The total volume of the assay was 1000 μL.

The rate laws of the reaction with respect to antioxidants and ABTS^•−^ were determined using the initial rate and the isolation methods. As soon as the antioxidants were added, the absorbance of ABTS^•−^ decreased.  Figure 1 displays the variation in absorbance during the first 7 min of the reaction.

The initial rates (d[A_0_]/dt) at different concentrations of an antioxidant or ABTS^•−^ were fitted to a kinetic law:

(1) $$\begin{array}{c} \frac{d\left[A_{0}\right]}{\text{dt}}=k{\left[{\text{ABTS}}^{•-}\right]}^{\alpha }{\left[\text{antioxidant}\right]}^{\beta }. \end{array}$$

The reaction orders were obtained by using logarithm expansion:

(2) $$\begin{array}{c} \log\left(\frac{d\left[A_{0}\right]}{\text{dt}}\right)=\log\;k+\alpha \;\log\left[{\text{ABTS}}^{•-}\right]+\beta \;\log\left[\text{antioxidant}\right]. \end{array}$$

In situations where ABTS^•−^ is in significant excess, the concentration of ABTS^•−^ can be considered constant. Therefore, *α* log [ABTS^•−^] remains constant [39]. The slope of the best-fit line is equivalent to *β*, which determines the order of the reaction with respect to the antioxidant. A similar approach is used to find the order of the reaction with respect to ABTS^•−^.  Table 4 lists the rate law and rate constant of each antioxidant reacting with ABTS^•−^. The order of the reactions with respect to each reactant is approximately one, except for the BSA/ABTS^•−^ reaction. It suggests a more complicated multistep mechanism could be involved in the reaction of BSA and ABTS^•−^.

### 3.4. Temperature Dependence of the Antioxidant Reactions

A temperature study provided the Arrhenius parameters for the antioxidant proteins and amino acids.  Table 4 summarizes the activation energy, pre-exponential factor A, and the temperature at which their peak activity was reached for each antioxidant reacting with ABTS^•−^.

According to the temperature study, the antioxidant activities of amino acids increase with the rise of temperature and peak around 49°C–55°C, followed by a gradual decrease to zero around 93°C. This is consistent with the literature report that Tyr and Trp are stable in the air at temperatures below 100°C [40, 41].

**Figure 2:**
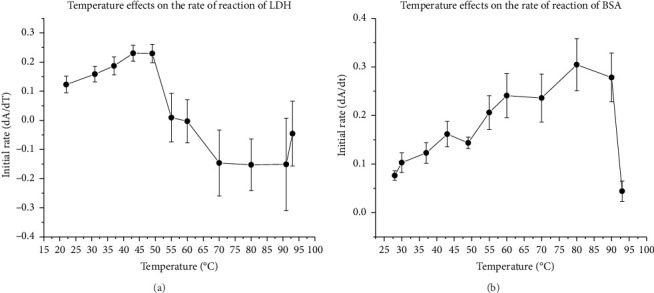
(a) Rate of reaction of LDH and ABTS^•−^ with respect to temperature under thermal stress with standard error bar. (b) Rate of reaction of BSA and ABTS^•−^ with respect to temperature under thermal stress with standard error bar.

LDH's antioxidant activity increased gradually with temperature until it peaked around 49°C. After that, it decreased drastically and eventually dropped to zero at around 55°C–60°C (Figure 2(a)). Above 50°C, LDH started to become cloudy, forming white precipitates and exhibiting negative measurements after 60°C. The large protein aggregates appeared to interact with UV light, causing light scattering and the unusual reading above the melting temperature. Those findings are consistent with literature stating that LDH melting temperature (the temperature at which 50% of the protein unfolds) is 50°C [21], and LDH can tolerate thermal stress until around 63°C [42].

As the temperature rises, BSA consistently increases its antioxidant activity until it reaches 80°C (Figure 2(b)). Interestingly, this peak temperature for BSA's activity is significantly higher than the temperature required for maximum activity for any amino acids or the reported melting temperature range for BSA, which is between 56°C and 69°C [43, 44].

Borzova et al. reported that as temperature increases, BSA unfolds and forms primary and secondary aggregates until 80°C when the large-sized aggregates are assembled [44]. As BSA unfolds, more antioxidant amino acid residues become accessible [45]. As a result, the overall antioxidant activity of BSA continued to increase after its melting temperature until 80°C, even though the antioxidant activity of each amino acid gradually decreased after 55°C.

Additionally, research has shown that BSA's disulfide bonds were reshuffled due to thermal denaturation [6], leading to more short-lived free SH groups that could react with the ABTS^•−^ radical. This reshuffling of disulfide bonds may also explain BSA's exceptional tolerance to thermal stress compared to LDH, which has no disulfide bonds present [46].

According to our data, all LDH Arrhenius parameters are similar to those of Trp, indicating that Trp may be the primary contributor to LDH's antioxidant activity. The reaction constant between BSA and Tyr is comparable, suggesting that Tyr may play a role in the protein's antioxidant activity. On the other hand, Arrhenius parameters of Cys are different from those of LDH or BSA. This suggests that Cys may be less critical than Trp and Tyr in the proteins' antioxidant properties.

Furthermore, as the temperature increased, the initial reaction rate of antioxidant activity in LDH or BSA increased 1.7 times slower than in Trp or Cys. However, it increased 1.6 times faster than in Tyr, indicating that combining Trp and Tyr may contribute to the proteins' antioxidant activity. Theoretically, more antioxidant residues may be exposed when protein unfolds with increasing temperature. However, the steric hindrance of antioxidant residues in LDH below the protein's melting temperature may still curb the antioxidant activity, as the data suggested. On the other hand, BSA's primary and secondary aggregation may lay open more Tyr and promote antioxidant activity as the temperature rises above the melting temperature. In both LDH and BSA, the formation of large aggregates rapidly diminished the antioxidant activities.

BSA has been used as a carrier and stabilizer for drugs, increasing the chance of the drug surviving in the GI tract [47, 48]. BSA's enhanced antioxidant activity at higher temperatures than its melting point can broaden its application in protecting therapeutic peptides and proteins from radical degradation and retaining the drug's activity during harvest, storage, and preparation. However, BSA's limitations as a drug carrier include its inability to translocate into cells other than through endocytosis, and its drug release also needs more study. For its use in nonpeptide or nonprotein drugs, it may impose additional care and cost.

### 3.5. The Effect of Radical Oxidation on LDH Enzymatic Activity

**Figure 3:**
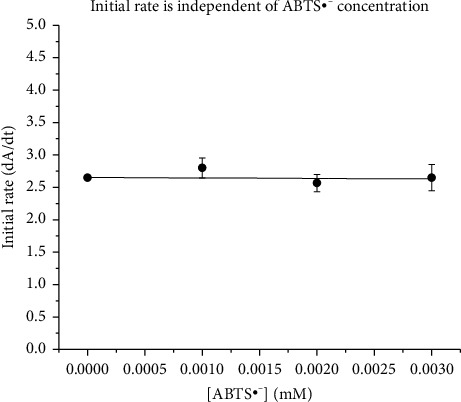
The initial rates of the enzyme reaction with respect to the ABTS^•−^ concentration with standard error bar. The graph shows that the presence of the radical did not affect the enzymatic activity of LDH.

We investigated the effect of ABTS^•−^ radical on the enzymatic activity of LDH. The ratio of radical to LDH was increased up to 47:1, and it was observed that the oxidation of the radical did not affect the LDH enzymatic activity for the conversion of lactate to pyruvate (as shown in Figure 3). This suggests that the reaction of antioxidant amino acids and ABTS^•−^ may occur on the protein's surface and may not significantly alter the protein's three-dimensional structure.

Furthermore, the enzymatic activities of LDH suggested that the LDH used in this study is in its native structure under our experimental condition. In addition, the results of rabbit and bovine LDH experiments at RT were consistent, indicating they have the same native structures.

LDH's tolerance to free radicals explains its dual function in the literature report, which states that LDH promotes cancer via its antioxidant properties and aids energy hijacking for cancer growth by transforming pyruvate into lactate, thereby providing energy for cancer growth [3, 27, 28]. Higher-than-normal LDH levels have been used as a biomarker for cancer and an indicator of ineffective cancer treatment [28, 49]. Its antioxidant role in cancer development could be further explored. Additionally, because it provides lactate as energy for cancer growth, LDH can be a therapeutic target for cancer treatment by developing inhibitors that reduce the production of lactate.

### 3.6. Reaction Mechanisms

#### 3.6.1. Mechanisms Based on the Kinetics

According to our rate law, an antioxidant amino acid Trp, Tyr, or Cys participated in a biomolecular reaction mechanism with one ABTS^•−^ molecule to produce an amino acid radical. Similarly, one LDH monomer seemed to quench only one ABTS^•−^ molecule. The similarity between LDH and Trp's kinetic parameters suggested that Trp could be the major contributor to LDH's antioxidant ability (refer to Tables 3 and 4).

In contrast, a BSA monomer could quench 0.6 ABTS^•−^ molecule (Table 4), and the similarity of reaction constants between BSA and Tyr suggested that Tyr may play a significant role in the protein's antioxidant ability. Other kinetic parameters showed that the reaction mechanism for BSA may be more complicated and multistepped.

Studies have proposed various free radical reaction mechanisms for different species, either the transfer of an electron or a hydrogen atom carrying an electron to ABTS^•−^ [50, 51]. Some studies have suggested a two-step reaction process, where a free radical is produced in the first step, followed by the self-quenching dimerization of the radicals [52–54].

Antioxidants uric acid and bilirubin share the common feature of –NH– on aromatic rings, and polyphenols have hydroxyls on the aromatic rings that can form resonance stabilized radicals. Rabie and colleagues also demonstrated the essential roles of –NH– on the indole and hydroxyl (–OH) on the aromatic ring in antioxidative activities [8, 9]. This agrees with our observations of the significant difference in antioxidant activity between Tyr and Phe, the latter missing an OH on the aromatic ring and losing antioxidant ability.

**Figure 4:**
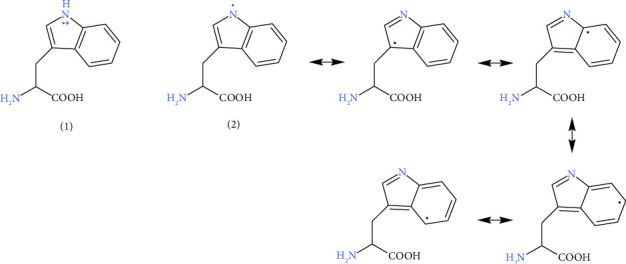
(1) and (2) are the two possible forms of Trp radicals. Both radicals are resonance stabilized, and the resonance structures for radical (2) are shown. Dimerization could happen to the combination of two radicals.

The indole or hydroxyl group on the aromatic ring of Trp and Tyr can donate an electron (see reaction (3) and amino acid radical product in Figure 4 (1)) or a hydrogen radical (see reaction (4), Figures 4 (2) and 5) to quench ABTS^•−^. The conjugated ring structure of Trp (or Tyr) allows for resonance structures after the indole (or hydroxyl) hydrogen atom is removed (Figure 4). A dimerization process may occur between two Trp (or Tyr) radicals to quench the amino acid radical (see reaction (5)). The neighboring Lys and Arg could also be potential candidates for radical reactions and dimerization.

(3) $$\begin{array}{c} {\text{ABTS}}^{•-}+e^{-}\text{ on antioxidant amino }{\text{acid}}^{*}⟶{\text{ABTS}}^{2-}+\text{amino }{\text{acid}}^{•+}, \end{array}$$

(4) $$\begin{array}{c} {\text{ABTS}}^{•-}+H^{•}\text{ on antioxidant amino }{\text{acid}}^{*}⟶{\text{ABTSH}}^{-}+\text{amino }{\text{acid}}^{•}, \end{array}$$

(5) $$\begin{array}{c} \text{Amino }{\text{acid}}^{•\left(+\right)}+\text{amino }{\text{acid}}^{•}⟶\text{amino acid }{\text{dimer}}^{\left(+\right)}, \end{array}$$

⁣^∗^The antioxidant amino acid could be free or on proteins.

Unlike Trp and Tyr, Cys has no conjugated ring structure. The reduced thiol (–SH) is likely to exhibit antioxidant activity in Cys [8, 9, 55]. Studies suggest that the donation of the hydrogen radical in free Cys amino acid occurs through a one-step H-atom transfer (HAT) due to the low bond dissociation enthalpies of the S–H bond and Cys's pH-independent properties [56, 57]. After this radical reaction, Cys may form disulfide bonds with another Cys molecule.

Based on our kinetic parameters, Cys did not appear to contribute to the antioxidant ability of BSA and LDH. BSA contains 35 Cys residues, but 34 are oxidized and form disulfide bonds with each other [7]. Only one Cys residue, Cys 34, is free and partially accessible on the surface of BSA. However, on average, freshly isolated BSA may contain less than one free SH group due to the reaction of Cys 34 on one BSA with Cys 34 on another BSA to form a dimer [41].

#### 3.6.2. Protein Docking and PyMOL Visualization of the Surface Tyr and Trp

The Chimera and AutoDock Vina tools were utilized to assess the potential binding sites on the surface of the receptor proteins. Through simulations, LDH or BSA proteins as receptors and ABTS^•−^ or AAPH peroxyl radicals as ligands would interact. Two sets of simulations were conducted: a general simulation to identify the area of the proteins with the highest affinity for the radicals and another to pinpoint regions of antioxidant activity based on surface-accessible Trp and Tyr residues. The protein docking simulations with lower binding scores are more stable and have a higher ligand affinity. The simulations revealed that the ABTS^•−^ radical binds to LDH most firmly in the interior pockets of the protein that can be accessed from the surface.

At a physiologically relevant pH of 7.4, ABTS^•−^ is an overall negatively charged radical with two negative sulfonates and one positive charge on the nitrogen on the thiazoline ring. Our calculation frequently showed its high affinity for positive surface Arg residues associated with the sulfonate groups on the ends of the ABTS^•−^ molecule through electrostatic forces and hydrogen bonding. Apart from Arg, His residues often formed hydrogen bonds between the ABTS^•−^ and LDH. The surface Lys residues occasionally formed hydrogen bonding with ABTS^•−^. Other residues found near ABTS^•−^ and the protein binding site were Val, Asn, Ala, Leu, Ile, and Gly. The hydrophobic amino acid side chains on Val, Ala, Leu, and Ile can attract the aromatic ring on ABTS^•−^ through hydrophobic interaction.

On the other hand, the AAPH peroxyl radical is positively charged. Val, Pro, His, Ser, Lys, Gly, Asn, and negatively charged Glu and Asp were all common in and near the interaction sites and some exhibited hydrogen bonds with the peroxyl radical in varying amounts between each trial. The scores of these trials were significantly higher than those between LDH and the ABTS^•−^ radical, indicating that the peroxyl radical interacts with LDH with less affinity than ABTS^•−^.

However, due to unproductive orientation, the most stable interactions between the LDH receptor and the respective ligands may not have antioxidant activity. This can happen when the radical ligand cannot collide with the protein receptor in a productive orientation due to steric hindrance from surrounding molecules. The nonproductive stable interaction visualized in the docking can trap the radicals, which may, in part, contribute to the experimentally observed reduction of the antioxidant activity of the amino acid in the protein compared to the free amino acids.

An LDH monomer comprises 331 amino acids in its primary sequence, which includes seven Tyr residues and six Trp residues. We used size-exclusion chromatography to determine the molecular weight of LDH in the phosphate buffer, which was around 150 kDa. This suggests that LDH exists as a tetramer in the solution. PyMOL visualization of the surface Tyr and Trp on Rabbit LDH tetramer shows that Tyr 82, Tyr 126, Tyr 144, Tyr 171, Tyr 238, Tyr 246, Tyr 280, Trp147, Trp 187, Trp 200, Trp 249, and Trp 323 are exposed to the surface at cutoff 10.0 square Angstroms. When the cutoff is increased to 15.0 and 20.0 square Angstroms, Tyr 126, Tyr 246, Tyr 238, Trp 147, Trp 187, and Trp 323 are exposed to the surface. The cutoff at 25.0 square Angstroms exposes only Tyr 126, Tyr 246, and Tyr 238, and no Trp. It was worth noticing that the antioxidant reactivity of Trp based on TE is about 2.5 times higher than Tyr. However, the larger surface exposure did not provide kinetic advantages or significant reactivities to these three Tyr. According to our protein docking experiments, Trp antioxidant characteristics dominated LDH. Trp 323, therefore, is the most possible reactive site on the protein.

**Figure 6:**
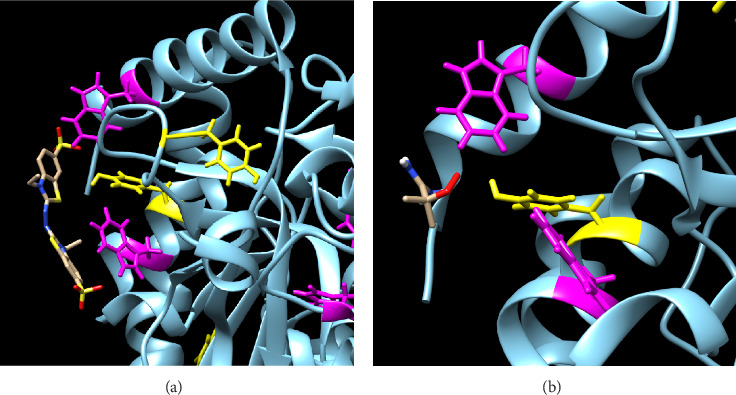
(a) Interaction between LDH chain D and ABTS^•−^. The proximity between Trp 323, Trp 147, and Tyr 144 on LDH (Trp shown in magenta and Tyr shown in yellow) presents a promising location for antioxidant activity. The ligand spans the entire triad, increasing the likelihood of an interaction displaying antioxidant ability. (b) Interaction between LDH and AAPH. The ligand interacts closely with Tyr 144 in the triad and is located near Trp 323 and Trp 147, indicating that this region could have promising antioxidant activity.

The AutoDock molecular modeling of LDH shows that productively oriented Trp or Tyr residues in proximity to one another on proteins are essential for their antioxidant activity. Especially when multiple large surface-accessible Trp or Tyr residues are located close to one another, they are more likely to form dimers and terminate the radicals. Targeted docking simulations of two radicals, ABTS^•−^ and AAPH peroxyl, showed that Trp 323, Trp 147, and Tyr 144 are the most likely sites on LDH to undergo antioxidant activity. Specifically, the reactive nitrogen on ABTS^•−^ came close to Trp 323 in the simulation, with Trp 147 and Tyr 144 residues in proximity to one another in space and a Lys 327 nearby to stabilize negatively charged sulfonate groups of ABTS^•−^ (Figure 6(a) and Supporting Figures 1A and 1B). The simulations suggest that dimerization between these residues is possible. Trp 323 may be more important for quenching ABTS^•−^, while Tyr 144 has a higher affinity to AAPH and may be more critical for reacting with AAPH peroxyl in the simulation (Figure 6(b)). These findings could have important implications for understanding the mechanisms of antioxidant proteins.

BSA is a protein with a molecular mass of 66,463 Da comprising 583 amino acids. It has three domains (I, II, III) [24, 58] and contains 20 Tyr residues and two Trp residues on the BSA monomer. PyMOL analysis of BSA showed no Trp exposure between the cutoff 6.0 to 25.0 square Angstroms, which agrees with our kinetic data that BSA resembles Tyr antioxidant activity. Among many other Tyr, Tyr 137 has a limited exposure at a cutoff of 6.0 square Angstroms. When the surface exposure cutoff was increased to 20.0 square Angstroms, Tyr 155, Tyr 160, Tyr 340, Tyr 400, and Tyr 451 were exposed.

**Figure 7:**
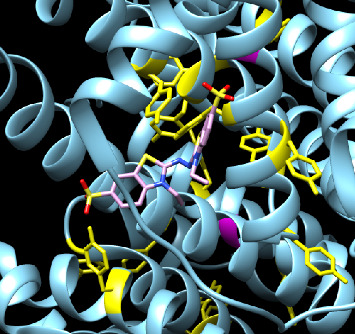
BSA monomer. There is a strong interaction between ABTS^•−^ and Tyr 137 and Tyr 160 on the BSA monomer. The docking score of −4.7 indicates that this is a relatively stable docking. This affinity shows promise for antioxidant activity because of the close interaction of ABTS^•−^ with Tyr residues and the possibility of forming a dimer to terminate the radicals due to the dense Tyr residues surrounding the area.

Protein docking further showed that Tyr 160, Tyr 155, and Tyr 137 are of considerable interest due to their surface exposure, proximity, and conducive orientation, which allow the formation of a Tyr dimer (Figure 7). In addition, adjacent Lys 159 could attract the negatively charged sulfonate groups of ABTS^•−^ radical. When the surface exposure cutoff was increased to 25.0 square Angstroms in PyMOL, only Tyr 451 was still exposed, with no other Tyr nearby to form dimers. Furthermore, Tyr 451 did not show favorable orientation for the radical reactions in our protein docking experiments despite being the most accessible and having a nearby Arg 194 that could attract the negatively charged sulfonate groups.

The docking experiments and their visualization showed that specific surface Trp and Tyr partially interact with the radicals due to steric hindrance and other surface physical properties. The experimental data echoed the molecular modeling and further suggested that the multiple surface Trp and/or Tyr residues only exhibited one equivalent free Trp antioxidant activity per LDH protein and 3.3 ± 0.5 free Tyr equivalent per BSA in the radial quenching.

LDH and BSA are predominantly helical structures with turns and loops on the surface. However, each protein's unique tertiary structure can affect its antioxidant ability through steric hindrance and physical properties of the protein surface; for instance, the charge distribution could attract or repel the radicals. The proximity of antioxidant residues in the tertiary structure can also affect antioxidant activity, increasing the likelihood of radical termination via dimerization.

## 4. Conclusions

Our study ranked the antioxidant activity of proteinogenic amino acids and proteins LDH and BSA. The kinetic data suggested a second-order biomolecular mechanism for the reaction of ABTS^•−^ with amino acid Trp, Tyr, Cys, or protein LDH. The antioxidant activity was temperature dependent. While Trp was responsible for LDH antioxidant activity, Tyr was essential for BSA antioxidant activity. Molecular computing revealed that the protein structures significantly constrained the antioxidant activities of the amino acids. The hydrophobic interaction and electrostatic forces of amino acid residues on the proteins could help recruit radicals to the reactive sites. A conducive microenvironment for antioxidant activities was multiple Trp and/or Tyr residues in proximity with one another in a productive orientation. While the current method of predicting antioxidant proteins is mainly based on their primary sequence, our research has demonstrated that examining the protein's tertiary and quaternary structure is crucial.

## Figures and Tables

**Figure 5:**
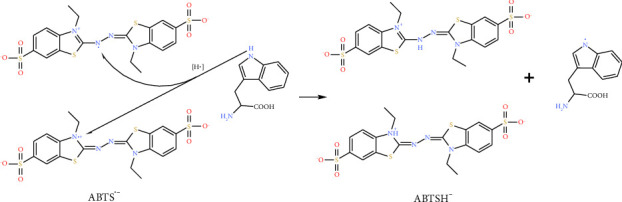
The reaction of ABTS^•−^ with Trp.

**Table 1:** Trolox equivalents (TEs) with standard deviation (SD) after 5-min inhibition with ABTS^•−^.

Antioxidant	TE (μmol TE/μmol) average (SD)	TE (μmol TE/g) average (SD)
BSA (monomer)	135 (18)	2030 (281)
Bov LDH (monomer)	106 (51)	2930 (1248)
Rab LDH (monomer)	107 (24)	3210 (576)
Tryptophan	101 (5.9)	509,000 (35,839)
Cysteine	30.5 (3.5)	237,000 (9139)
Tyrosine	38.7 (11)	213,000 (58,937)
Lysine	0.193 (0.0021)	1320 (15.5)
Arginine	0.0325 (0.0041)	186 (23.3)
Valine	0.0280 (0.00021)	238 (2.12)
Histidine	0.00689 (0.0028)	44.4 (18.5)
Leucine	0.0056 (0.00021)	43 (1.41)

**Table 2:** ORAC average Trolox equivalent.

Antioxidant	TE (μmol TE/μmol) protein (SD)	TE (μmol TE/g) protein (SD)
BSA (monomer)	19 (1.7)	285 (19)
Bov LDH (monomer)	8.9 (0.99)	262 (29)

**Table 3:** Summary of EC50s for LDH, BSA, and amino acids with noticeable antioxidant activities and the orders of the reaction with respect to the antioxidants.

Antioxidant	Order of the reaction	Average EC50 (SD) at 1 min (μmol/L)	Average EC50 (SD) at 5 min (μmol/L)	Average EC50 (SD) at 7 min (μmol/L)
Bov LDH (monomer)	1	10.6 (0.32)	4.79 (1.09)	4.21 (1.07)
Rab LDH (monomer)	0.9	10.8 (0.83)	4.90 (1.21)	4.47 (1.31)
BSA (monomer)	1	9.98 (1.55)	4.69 (0.88)	3.92 (0.86)
Tryptophan	1	11.6 (4.13)	4.50 (0.20)	3.94 (0.18)
Cysteine	1	11.0 (0.46)	10.5 (0.24)	9.96 (0.21)
Tyrosine	0.9	38.9 (2.75)	13.8 (3.76)	11.2 (6.37)
Lysine	1	3520 (28)	1980 (42)	1450 (35)
Arginine	1	20,900 (71)	15,100 (495)	12,500 (707)
Histidine⁣^∗^	0.8	131,000	50,700	41,500
Valine	0.8	164,000 (1410)	59,600 (1840)	49,400 (1480)
Leucine	0.5	228,000 (11,300)	76,800 (495)	65,100 (424)

⁣^∗^Only one dataset is available for histidine.

**Table 4:** Summary of the kinetic study and temperature for peak antioxidant activities.

Antioxidant	Order (SD) of the reaction with respect to antioxidant	Order (SD) of the reaction with respect to ABTS^•−^	Reaction constant (SD)	Activation energy (J/mol)	Pre-exponential factor (A/n)	Peak activity temperature (°C)
LDH	0.95 (0.08)	0.96 (0.33)	62,754 (8708)	19,619.4	1.91 × 10^8^	50
BSA	1.01 (0.06)	0.57 (0.03)	2055.4 (145.8)	21,333.7	1.25 × 10^7^	80
Tryptophan	0.99 (0.16)	0.94 (0.04)	63,065 (12,970)	19,827.2	2.1 × 10^8^	55
Tyrosine	0.90 (0.02)	0.96 (0.02)	2244.7 (166.9)	10,262.8	1.47 × 10^5^	55
Cysteine	1.02 (0.06)	1.05 (0.14)	274,228 (14,938)	2484.97	7.45 × 10^5^	49

## Data Availability

The data used to support the findings of this study are included within the article.

## Supporting Information

Supporting Figure 1A and Supporting Figure 1B are included.
