# Supplementary material for: Potential Mechanisms of Lactate Dehydrogenase and Bovine Serum Albumin Proteins as Antioxidants: A Mixed Experimental–Computational Study
Source: Biochem Res Int. 2025 Feb 10;2025:9638644. doi: 10.1155/bri/9638644 (PMC11832265; doi:10.1155/bri/9638644)
Supplement: Supporting Information — Additional supporting information can be found online in the Supporting Information section. [file 9638644.f1.docx]

ABTS^·-^ moves around Trp 323, enabling the redox reaction

Supplementary Figure 1A

Supplementary Figure 1B
